# Response to the Svingen Comments on Li *et al*. Effects of *in Utero* Exposure to Dicyclohexyl Phthalate on Rat Fetal Leydig Cells. *Int. J. Environ. Res. Public Health*, 2016, *13*, 246

**DOI:** 10.3390/ijerph13060533

**Published:** 2016-05-25

**Authors:** Xiaoheng Li, Xiaomin Chen, Guoxin Hu, Linxi Li, Huina Su, Yiyan Wang, Dongxin Chen, Qiqi Zhu, Chao Li, Junwei Li, Mingcang Wang, Qingquan Lian, Ren-Shan Ge

**Affiliations:** 1 The Second Affiliated Hospital & Yuying Children’s Hospital of Wenzhou Medical University, Wenzhou 325027, China; yaoyao_0111@126.com (X.L.); xminch@163.com (X.C.); lilinxi1234@163.com (L.L.); suhuina1988@163.com (H.S.); wyy19880119@163.com (Y.W.); gerenshan2@163.com (D.C.); flower3377@163.com (Q.Z.); dishboy@163.com (C.L.); 2 Department of Pharmacology, School of Pharmaceutical Sciences, Wenzhou Medical University, Wenzhou 325000, China; hgx@wzmc.edu.cn (G.H.); gravity172@gmail.com (J.L.); 3 Taizhou Hospital of Zhejiang Province, Taizhou 317000, China; wangmc@enzemed.com

Referring to the comments of Svingen [1] on our latest publication about *Effects of in utero Exposure to Dicyclohexyl Phthalate on Rat Fetal Leydig Cells* [2], we would like to give some comments.

In general, we agree with Svingen that attention should be paid to cellularity when interpreting quantitative expression data in tissues, otherwise important information may be overlooked or wrongly extrapolated. Many studies have demonstrated that phthalates act as novel antiandrogens and suppressed insulin-like 3 expressions [3,4,5,6,7,8,9,10,11,12,13]. The developmental effects of phthalates as antiandrogens in androgen-dependent tissues include the shortening of anogenital distance (AGD), hypospadias, cryptorchidism, and reduced weights of seminal vesicles, epididymis and prostate as well as retention of nipples [11,14,15]. The collection of these malformations in the male reproductive tract is referred as the “phthalate syndrome”. The AGD is regulated in the androgen-dependent manner: the skin between genital papillar and the anus is regulated by androgens in the prenatal stage. Because of androgen action, the AGD in male pups is about twice that of females [16,17,18]. In the rat, androgen is critical for the development of the male reproductive phenotype. Deficiency of androgen causes the formation of the female phenotype [19]. Testosterone produced by fetal Leydig cells is very important for differentiation of the Wolffian ducts, sex accessory tissues, and testicular descent [19]. In the rat, the biosynthesis of testosterone requires at least four critical enzymes, cytochrome P450 cholesterol side chain cleavage enzyme (CYP11A1), 3β-hydroxysteroid dehydrogenase 1 (3β-HSD), cytochrome P450 17α-hydroxylase/20-lyase (CYP17A1), 17β-hydroxysteroid dehydrogenase 3 (17β-HSD3) [20]. In the peripheral tissues, testosterone can be metabolized into a more potent metabolite, dihydrotestosterone, by catalysis of 5α-reductase 1 (SRD5A1). Dihydrotestosterone is also very important for development of external genital and the prostate [21]. Besides androgen, the fetal Leydig cells produce another hormone, INSL3, which is critical for the initial testis descent [22].

When we explore the mechanism of an endocrine disruptor, for example, a phthalate, as an antiandrogen, several factors must be paid attention to: antagonism against the androgen receptors, blockade in the androgen biosynthesis, suppression of the androgen metabolic activation (e.g., by 5α-reductase), down-regulation of steroidogenic enzymes, reduced fetal Leydig cell numbers, and delay of fetal Leydig cell differentiation.

Some phthalates, such as di-*n*-butyl phthalate, directly bind to the rat androgen receptor as antagonists [23]. When the [^3^H]R1881, an androgen receptor agonist, was used to bind to the androgen receptor and its relative binding affinity was set as 100, the relative binding affinity for dihydrotestosterone, testosterone, diethyl phthalate, butylbenzyl phthalate, and di-*n*-butyl phthalate were 137.9008, 19.1768, 0.0004, 0.0085, and 0.0112, respectively [23]. Some phthalates, such as dicyclohexyl phthalate (DCHP), directly inhibit the activities of several androgen biosynthetic enzymes [24]. The Ki values of DCHP for inhibiting human and rat testis 3β-HSD activities are 16.98 and 16.47 μM, and those of DCHP for human and rat 17β-HSD3 activities are 5.49 and 8.48 μM, respectively [24].

In the most cases, phthalates down-regulate the gene expression levels of steroidogenesis-related genes, such as Star, Cyp11a1, and Hsd3b1, as well as Insl3. When the expression levels of these genes are measured in the fetal testis and are presented, the cellularity should be highlighted as the factor to be considered. Testis is organ that contains many different cell types, Leydig cells, peritubular myoid cells, Sertoli cells, germ cells, and immune cells. Therefore, to explore whether a chemical affects the expression level of a certain gene, the fetal Leydig cells should be enumerated and the expression levels of these genes may be adjusted by the number of fetal Leydig cells. Indeed, Svingen agrees with us that the number of fetal Leydig cells was enumerated in the current research paper [10], in which we revealed that DCHP affected fetal Leydig cell morphology and caused them to aggregate but did not change their numbers [10]. Therefore, the reduced expression levels of Star, Hsd3b1 and Insl3 are possibly caused by the direct effects of DCHP on their transcription.

Svingen mentions that most of the genes examined were downregulated by higher doses of DCHP and only Cyp11a1 was not affected, suggesting that an alternative mode of action: possibly affecting the differentiation of fetal Leydig cells. Svingen suggested that we might check additional biomarkers of fetal Leydig cells such as Delta-like 1 to show whether these fetal Leydig cells are immature cells. It is true that some immature Leydig cell biomarkers such as Delta-like 1 can show whether these fetal Leydig cells are immature or mature. However, many phthalates, including di-*n*-butyl [25], dipentyl [26], dihexyl [26], diheptyl [26], diisononyl [26], and di-(2-ethylhexyl) [10] phthalate, actually down-regulated Cyp11a1, although DCHP did not inhibit Cyp11a1 even very higher dose was used [10]. Unlike the adult Leydig cells, the second generation of Leydig cells, during puberty, the developmental stages of fetal Leydig cell generation was not well characterized. For adult Leydig cell generation, we well characterized the developmental stages with clear morphological changes, biochemical biomarkers and functional features. The developmental stages of adult Leydig cells are conceptually divided into four stages: stem, progenitor, immature, and adult Leydig cells [20]. Stem Leydig cells are spindle-shaped, with no biomarkers in the Leydig cell lineage such as steroidogenic enzymes and Star and do not produce any steroids [27]. They contain stem cell biomarkers such as platelet-derived growth factor receptor α [27], nestin [21] and CD90 [28], and they are abundant in the testis in the neonatal period [27]. Progenitor Leydig cells are also spindle-shaped and morphologically are similar to the stem Leydig cells [29,30] and are developed around postnatal day 12–14 in the rat testis and express several steroidogenic enzymes such as CYP11A1, 3β-HSD1, CYP17A1, SRD5A1, and AKR1C14 but lack the final step enzyme 17β-HSD3, thus producing predominantly androsterone [31]. Around day 35 postpartum, immature Leydig cells are developed and these cells are round, have many lipid droplets [29,30], not only contain all these enzymes like progenitor Leydig cells but also have the 17β-HSD3, thus primarily producing 5α-androstane-3α,17β-diol [31]. Adult Leydig cells are large and round cells [29,30], and also contain androgen biosynthetic enzymes but have no expression of SRD5A1, thus mainly secreting testosterone [31]. Morphologically, adult Leydig cells have no lipid droplets [29]. Before the different cell types in the fetal Leydig cell lineage are well characterized, it is difficult to distinguish between the effects of DCHP on the development of fetal Leydig cells or direct down-regulation of these genes in mature fetal Leydig cells. It seems that DCHP at least partially affects the development of fetal Leydig cells, because we identified that the fetal Leydig cells in DCHP-treated testis had small cell size and cytosolic volume [10]. In the future, when more information about developmental changes of fetal Leydig cells is added, the effects of a chemical on the development of fetal Leydig cells can be well defined. In summary, cellularity and developmental changes may be considered when we explore the expression levels of certain genes in the fetal testis as the case of DCHP *in utero* exposure.
